# Impact of Maxwell velocity slip and Smoluchowski temperature slip on CNTs with modified Fourier theory: Reiner-Philippoff model

**DOI:** 10.1371/journal.pone.0258367

**Published:** 2021-10-14

**Authors:** Tanveer Sajid, Wasim Jamshed, Faisal Shahzad, M. A. Aiyashi, Mohamed R. Eid, Kottakkaran Sooppy Nisar, Anurag Shukla

**Affiliations:** 1 Department of Mathematics, Capital University of Science and Technology (CUST), Islamabad, Pakistan; 2 Department of Mathematics, College of Science, Jazan University, Jazan, Saudi Arabia; 3 Department of Mathematics, Faculty of Science, New Valley University, Al-Kharga, Al-Wadi Al-Gadid, Egypt; 4 Department of Mathematics, Faculty of Science, Northern Border University, Arar, Saudi Arabia; 5 Department of Mathematics, College of Arts and Sciences, Prince Sattam bin Abdulaziz University, Wadi Aldawaser, Saudi Arabia; 6 Department of Applied Science, Rajkiya Engineering College Kannauj, Kannauj, India; Tongji University, CHINA

## Abstract

The present article presents a novel idea regarding the implementation of Tiwari and Das model on Reiner-Philippoff fluid (RPF) model by considering blood as a base fluid. The Cattaneo-Christov model and thermal radiative flow have been employed to study heat transfer analysis. Tiwari and Das model consider nanoparticles volume fraction for heat transfer enhancement instead of the Buongiorno model which heavily relies on thermophoresis and Brownian diffusion effects for heat transfer analysis. Maxwell velocity and Temperature slip boundary conditions have been employed at the surface of the sheet. By utilizing the suitable transformations, the modeled PDEs (partial-differential equations) are renewed in ODEs (ordinary-differential equations) and treated these equations numerically with the aid of bvp4c technique in MATLAB software. To check the reliability of the proposed scheme a comparison with available literature has been made. Other than Buongiorno nanofluid model no attempt has been made in literature to study the impact of nanoparticles on Reiner-Philippoff fluid model past a stretchable surface. This article fills this gap available in the existing literature by considering novel ideas like the implementation of carbon nanotubes, CCHF, and thermal radiation effects on Reiner-Philippoff fluid past a slippery expandable sheet. Momentum, as well as temperature slip boundary conditions, are never studied and considered before for the case of Reiner-Philippoff fluid past a slippery expandable sheet. In the light of physical effects used in this model, it is observed that heat transfer rate escalates as a result of magnification in thermal radiation parameter which is 18.5% and skin friction coefficient diminishes by the virtue of amplification in the velocity slip parameter and maximum decrement is 67.9%.

## 1. Introduction

Since 1991, this is the subject of the inquiry because of its synthetic and basic existence since the beginnings of the era of carbon nanotubes. In several technical and industrial applications such as electrochemical super-capacitors, transistors, sensors and field emitting machines, engine transmission oil, heat exhaust flue gas recovery, electrical cooling, nuclear cooling, nanofluid boiling, etc. special properties such as high surface radiation, carbon nanotubes (CNTs) were studied. These applications got the researcher’s attention to work on CNTs. Kundu et al. [1] presented a carbon nanotube-based superior convective flow of Maxwell nanoliquid over an expanding sheet embedded through multiple slip influences. Al-Hanaya et al. [2] inspected the impact of single and multiwall carbon nanotubes on micropolar fluid along an extendable surface. Hayat et al. [3] published a computational approach on diffusion-species of carbon nanotubes. Nagalakshm et al. [4] pondered the magnetohydrodynamic (MHD) radiative flow of an incompressible steady flow of Carreau nanofluid along with carbon nanotubes affecting through a nonlinear elongated plate. Muhammad et al. [5] established the mathematical scheme concerning a Casson fluid through a stretching surface along in existence of CNTs, thermal non-linear radiation, and heat sink (source). Khalid et al. [6] pondered the impression of MHD and CNTs on fluid movement through an expandable plate. Hosseinzadeh et al. [7, 8] investigated cross-fluid flow past a cylinder and rotate cone with the inclusion of nanoparticles and motile gyrotactic microorganisms. Lu et al. [9] analyzed the behaviour of nanoliquids containing CNTs along with a CCHF and entropy production. The impact of carbon nanotubes on mixed convective radiative liquid flowing past a nonlinearly elongated sheet along with slip constraint is inspected by Mandal et al. [10]. Chaudhary et al. [11] elaborated the viscous nanofluid flowing with electrical conductivity close to a stagnating area of CNTs in water via extending surface. Researchers have employed the law of conduction suggested by Fourier to evaluate the heat transport analysis of liquid. Research investigations [12–15] probed the impacts of CNTs, MoS_2,_ and Fe_3_O_4_ hybrid nanoparticles with the consideration of different base fluids moving over diverse surfaces. Cattaneo [16] modified the Fourier law of conduction by inserting the relaxation time phenomenon for the heat-flux and later on Christov [17] introduced a derivative prototypical of Cattaneo’s rule and that developed known as Cattaneo-Christov law having immense applications in the engineering and medical field like nuclear reactor cooling, pasteurization of milk, electronic devices, hybrid power generators, etc. Based on these applications Akbar et al. [18] discussed the magnetic field and CCHF effects on CNTs-water nanofluid moving via an expandable plate. Ali et al. [19] pondered the characteristics of Cattaneo-Christov diffusion model for the flow of stagnancy point of Carreau nanofluid along an extendable surface embedded with chemical reaction effect. Ibrahim et al. [20] checked the flux impact of Cattaneo-Christov on free convective incompressible viscous fluid flowing through a vertical plate along with viscous dissipation and thermal radiation. Rasool et al. [21] researched to disclose the features of the inhomogeneous induced magnetical impact of nanoliquid flowing manifested with CCHF effect via an exponential extendable sheet. Khan et al. [22] scrutinized the impact of three different types of nanoparticles like *Al*_2_*O*_3_, *TiO*_2_ and copper *Cu* by considering *H*_2_*O* as a standard liquid through a nonlinearly expandable plate embedded with the CCHF. Shah et al. [23] investigated the effect of carbon nanotubes and CCHF on three-dimensional rotating liquid past an extendable plate. In the thermal radiation incidence model of the Cattaneo-Christov system, Dogonchi et al. [24] studied the heat transport analyzes of nanofluid flow between two parallel plating model.

Thermal radiation is one of three processes that permit energy exchange for bodies with different temperatures. The electro-magnetic wave emission from substances is characterized by thermal radiation (variation of its interior energy). It transmits radiation from ultraviolet to far-field infrasound depending on the material’s temperature. Thermal radioactivity is utilized where the high-temperature variance is essential having immense utilization in the industry like thermal furnaces, nuclear reactors, polymer production, combustion reactors, rubber production, etc. Sreedevi and Reddy [25] pondered the impact of thermal radiation on multi-wall carbon nanotube with kerosene as a base fluid past an extendable surface and found that the temperature field escalates owing to an increment in the thermal radiation parameter. The thin-film stream of a viscous nanofluid is introduced by Shah et al. [26] over a horizontal, spinning disk immersed in thermal radiative effect. Reddy et al. [27] inspected the influence of thermal radiative flow and viscous dissipative on RPF via a porous medium. Khan et al. [28] probed the impacts of thermophoretic and thermal radiative flow on second-grade two-dimensional magneto-liquid in which viscous dissipative is introduced via a stretchable surface. Hosseinzadeh et al. [29] scrutinized the influence of thermal radiation and viscous dissipation on hybrid nanoparticles over the vertical cylinder with the consideration of different shapes of nanoparticles. Kumar et al. [30] explored the effect of viscous dissipative and Joule effect on 3-dimensional nanofluid flow of Jeffrey model past an extending sheet underneath the influence of thermal exponential radiation. Makinde et al. [31] deliberated the influence of thermal radiative and melting heat transfer on magneto micropolarity liquid through an elongated sheet. The influence of thermal radiative flow and viscous dissipative on water squeezing among two Riga surfaces is scrutinized by Ahmed et al. [32]. The impact of nanoparticles, microorganisms, and thermal radiation on bioconvection past a magnetic field has been debated in detail by Hosseinzadeh et al. [33]. Hybrid nanoparticles fluid flow through an octagonal porous medium with the inclusion of MHD and thermal radiation are scrutinized by Hosseinzadeh et al. [34]. Rashed et al. [35] observed the effect of thermal radiative flow and magnetohydrodynamics over a porous rotating infinite disk together with Soret and Dufour effects.

The slip-free effect happens where the fluid and the wall are equal in velocity. On the other hand, the state in which the fluid and the wall velocity are different is called the slip velocity. In the case of temperature slip of the fluid and the sheet surface temperature is different. The liquid impact of boundary slip has distinguished utilizations like improving artificial interior cavities and valves of the heart. Shafiq et al. [36], under the simultaneous influence of thermal slip and the convective boundary conditions, examined revolving frame fluid flow. In presence of slip boundary conditions, Mukhopadhyay [37] conducted a thorough analysis of the unstable convective boundary layer flow and the heat transfer over the vertical stretch field. Thanks to the increase of the speed slip parameter the rate of heat transfer decreases. For the thermal slip parameter, the same function is noted also. Raza et al. [38] investigated thermal radiation and slip velocity effects concurrently with a heated convective stretching plate. Abbas et al. [39] exhibited the flow of hybrid nanoparticles with slip velocity and MHD. In the presence of thermal and hydrodynamic slip conditions, Rao et al. [40] proposed the laminar free convective boundary-layer flow of a Casson viscoelastic fluid external to a vertical permeable spherical cone. The results of the thermal and velocity boundary conditions on thermally radiative ferrofluid motions over a flat plate are studied by Sejunit and Khaleque [41]. Li and Keh [42] discussed analytically the thermophoretic displacement of the spherical particles at the core of a gaseous material in the specified temperature gradient. Sarabandi and Moghadam [43] analyzed the steady-state laminar flow of non-Newtonian fluid in a circular microchannel embedded with slip velocity condition.

The reasons behind the organization of this research are given below:

■ The Maxwell velocity and Smoluchowski temperature boundary slip constraints are considered to study a slip effect on RPF which is not explored yet in available literature. Before this investigation, no literature is available to study the effect of Maxwell velocity and Smoluchowski temperature jump boundary conditions on the Non-newtonian fluid past a stretching sheet.
■ Other than Buongiorno nanofluid no literature is available to study the effect of nanoparticles on Reiner-Philippoff fluid past a stretching sheet. Attempt has been made in this article to implement Tiwari and Das nanofluid model for the case of heat transport analysis of Reiner-Philippoff model with blood as a standard fluid.
■ Before this investigation, no literature on Reiner-Philippoff fluid is available to study heat flux of Cattaneo-Christov scheme along with thermal radiation is used to investigate the heat transfer analysis. Thermal radiation present in the fluid amplifies the heat transfer rate. The main purpose is to investigate the heat transfer rate with the inclusion of CCHF and thermal radiation.
■ SWCNT/MWCNT are never used before for the case of Riner-Philippoff fluid.

## 2. Flow model

The expression Reiner-Philippoff [27] of stress deformation is given:

$$\frac{\partial u}{\partial y}=\frac{\tau }{\mu_{\infty }+\frac{\mu_{0}-\mu_{\infty }}{1+{\left(\frac{\tau }{\tau_{s}}\right)}^{2}}}.$$ (1)

The symbols *τ*, *τ*_*s*_, *μ*_0_ and *μ*_∞_ indicates shear-stress, reference shear-stress, zero shear viscosity, and limited viscosity. Reiner-Philippoff model responds like shear thinning, Newtonian, and shear thickening subjected to a variation in fluid viscosity. Mathematical expression regarding RPF flow function is written as

$$f(\sigma )=\frac{\sigma }{1+\frac{\lambda -1}{1+\sigma^{2}}},$$ (2)

whereas $\sigma =\frac{\tau }{\tau_{s}}$ and $\lambda =\frac{\mu_{0}}{\mu_{\infty }}$. The expression *λ* in fluid flow function represents Reiner-Philippoff fluid parameter. Reiner-Philippoff behaviour is Newtonian for *λ* = 1, shear thickening *λ*<0, and shear thinning *λ*>1.

**Fig 1:**
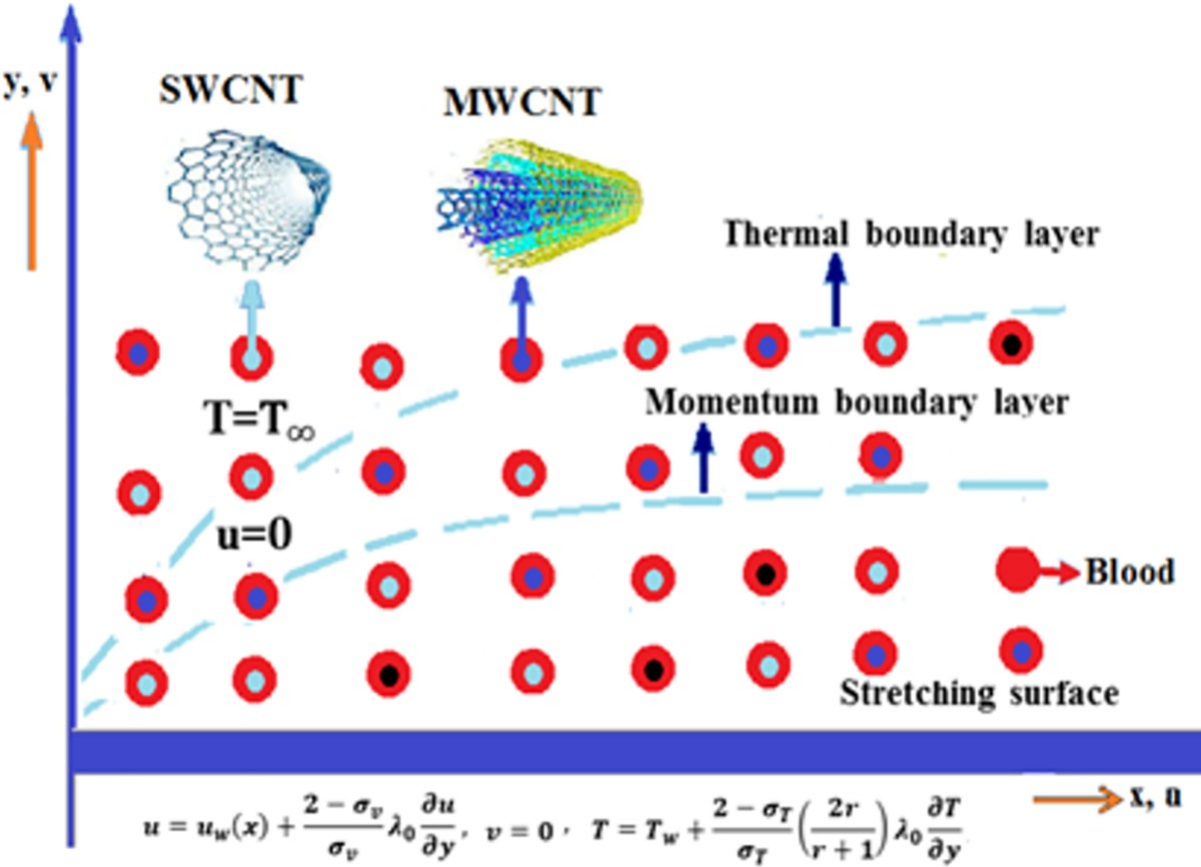
Physical description of flow model.

**Fig 2:**
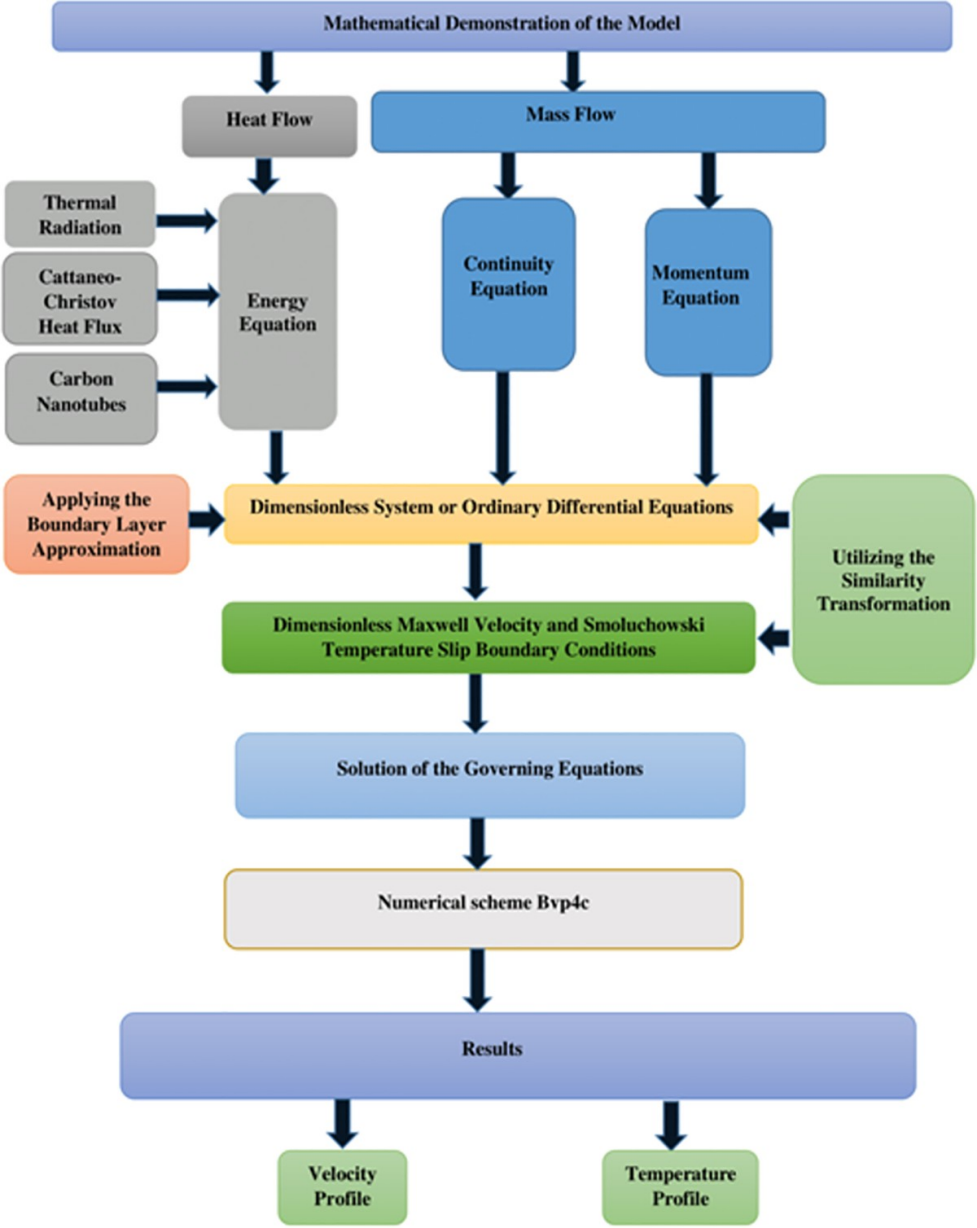
Mathematical model’s flow chart structure.


Fig 1 is designed to reflect the physical interpretation of RPF impinging on an expanding surface having an extending velocity *u*_*w*_ influential lengthways *x*−direction. The symbols *T*_0_ and *T*_∞_ depicts the fluid energy and concentration at the surface and ambient temperature outlying from the surface. The momentum equation has been scrutinized with the inclusion of carbon nanotubes. Momentum and thermal boundary layers occur when the velocity and temperature of the fluid is 0.99% of the free stream fluid velocity as well as temperature. Under these boundary conditions, the viscous, as well as velocity effects, hold. Heat transfer analysis has been carried out with the inclusion of solar thermal thermal and CCHF. Radiation is one of the best sources of heat and requires no medium for its propagation. Heat transfer rate in the case of the temperature equation is improved with the inclusion of carbon nanotubes, CCHF, and thermal radiation. Momentum and temperature slip boundary conditions have been employed at the boundary surface by the consideration of the assumption that the surface is rough, wet, and the effect of the adhesive forces is negligible. The fluid is incompressible and laminar. The density, as well as pressre, is constant. Human blood is considered as a base liquid flowing through a stretchable surface with the inclusion of SWCNT and MWCNT. The density *ρ*_*nf*_, thermal diffusion *α*_*nf*_, nanofluid specific heat (*ρC*_*p*_)_*nf*_, thermal conductance *k*_*nf*_ are specified by the mathematical expressions mentioned below [1–3]. Fig 2 demonstrates the flow chart of a mathematical model.

$$\begin{array}{l} \rho_{nf}=(1-\phi )\rho_{f}+\phi \rho_{CNT},\\ \mu_{nf}=\frac{\mu_{f}}{1-\phi_{2.5}},\\ \alpha_{nf}=\frac{k_{nf}}{(\rho C_{p}{)}_{nf}},\\ (\rho C_{p}{)}_{nf}=(1-\phi )(\rho C_{p}{)}_{f}+\phi (\rho C_{p}{)}_{CNT},\\ \frac{k_{nf}}{k_{f}}=\frac{(1-\phi )+2\phi \frac{k_{CNT}}{k_{CNT}-k_{f}}ln\frac{k_{CNT}+k_{f}}{2k_{f}}}{(1-\phi )+2\phi \frac{k_{f}}{k_{CNT}-k_{f}}ln\frac{k_{CNT}+k_{f}}{2k_{f}}}, \end{array}\}.$$ (3)

Table 1 displays the thermo-physical features of essential fluid and CNTs below [6].

**Table 1:** Thermo-physical characteristics.

Property	Human Blood (38°C)	SWCNT	MWCNT
*C*_*p*_	3594	425	796
*ρ*	1053	2600	1600
*k*	0.492	6600	3000
*Pr*	24	-	-

The models of the continuity, momentum, and energy equations are described below [27]:

$$\frac{\partial u}{\partial x}+\frac{\partial v}{\partial y}=0,$$ (4)

$$u\frac{\partial u}{\partial x}+v\frac{\partial u}{\partial y}=\nu_{nf}\frac{\partial \tau }{\partial y},$$ (5)

$$u\frac{\partial T}{\partial x}+v\frac{\partial T}{\partial y}+\lambda_{E}\Phi_{E}=\frac{k_{nf}}{(\rho C_{p}{)}_{nf}}\frac{\partial^{2}T}{\partial y^{2}}-\frac{1}{(\rho C_{p}{)}_{nf}}\frac{\partial q_{r}}{\partial y},$$ (6)

The physical boundary conditions (BCs) are [27]:

$$\begin{array}{l} y=0:u(x,y)=ax^{\frac{1}{3}}+\frac{2-\sigma_{v}}{\sigma_{v}}\lambda_{0}x^{\frac{1}{3}}\frac{\partial u}{\partial y},v=0,T=T_{0}+\frac{2-\sigma_{T}}{\sigma_{T}}\left(\frac{2r}{r+1}\right)\frac{\lambda_{0}}{Pr}x^{\frac{1}{3}}\frac{\partial T}{\partial y}\\ y\to \infty :u\to 0,T\to T_{\infty }, \end{array}\}$$ (7)

where CCHF [19–22] is given by

$$\Phi_{E}=\left(u^{2}\frac{\partial^{2}T}{\partial x^{2}}+v^{2}\frac{\partial^{2}T}{\partial y^{2}}+2uv\frac{\partial^{2}T}{\partial x\partial y}\right)+\left((u\frac{\partial u}{\partial x}+v\frac{\partial u}{\partial y})\frac{\partial T}{\partial x}+(u\frac{\partial v}{\partial x}+v\frac{\partial v}{\partial y})\frac{\partial T}{\partial y}\right).$$ (8)

The expression of heat radiation flux [32] is specified as

$$q_{r}=-\frac{4\sigma^{*}}{3\kappa^{*}}\frac{\partial T^{4}}{\partial y}=-\frac{16\sigma^{*}}{3\kappa^{*}}T^{3}\frac{\partial T}{\partial y}.$$ (9)

The function of a stream is defined as $u=\frac{\partial \psi }{\partial y}$ and $v=\frac{-\partial \psi }{\partial x}$. By utilizing the appropriate similarity transformation [27] with velocity $U(x)=ax^{\frac{1}{3}}$ given below

$$\psi =\sqrt{a\nu }x^{\frac{2}{3}}f(\eta ),\eta =\sqrt{\frac{a}{\nu }}x^{\frac{-1}{3}}y,\tau =\rho \sqrt{a^{3}\nu }g(\eta ),\theta (\eta )=\frac{T-T_{\infty }}{T_{0}-T_{\infty }},\}$$ (10)

the Eqs (4)–(6) are transformed into non-dimensionless ODEs

$$g\prime =\frac{A_{1}}{3}f^{\prime 2}-\frac{2}{3}ff\prime \prime ,$$ (11)

$$g=f\prime \prime \frac{g^{2}+\lambda \gamma^{2}}{g^{2}+\gamma^{2}},$$ (12)

$$\left(A_{2}+\frac{4}{3}Rd\right)\theta \prime \prime +\frac{2}{3}A_{3}Prf\theta \prime -Pr\delta (ff\prime \theta \prime +\eta f^{2}\theta \prime \prime )=0,$$ (13)

associated boundary conditions are

$$\begin{array}{l} \eta =0:f(\eta )=0,f\prime (\eta )=1+\gamma_{1}f\prime \prime ,\theta (\eta )=1+\gamma_{2}\theta \prime ,\\ \eta \to \infty :f^{\prime }(\eta )\to 0,\theta (\eta )\to 0. \end{array}\}$$ (14)

The associated non-dimensionless quantities are defined below

$$\begin{array}{l} \gamma =(\frac{\tau_{s}}{\rho \sqrt{a^{3}v}}),\lambda =\frac{\mu_{0}}{\mu_{\infty }},Pr=\frac{\mu C_{p}}{k_{\infty }},R_{d}=\frac{16\sigma T_{\infty }^{3}}{3k^{*}k_{\infty }},\delta =a\lambda_{E}\\ \gamma_{1}=\frac{2-\sigma_{v}}{\sigma_{v}}\lambda_{0}\sqrt{\frac{a}{v}},\gamma_{2}=\frac{2-\sigma_{T}}{\sigma_{T}}\left(\frac{2r}{r+1}\right)\frac{\lambda_{0}}{Pr}\sqrt{\frac{a}{v}}, \end{array}\}$$ (15)

where *A*_1_, *A*_2_ and *A*_3_ are given by

$$\begin{array}{ll} A_{1} & =\frac{1}{(1-\phi )+\phi \frac{\rho_{CNT}}{\rho_{f}}},\\ A_{2} & =\frac{k_{nf}}{k_{f}},\\ A_{3} & =(1-\phi )+\phi \frac{(\rho C_{p}{)}_{CNT}}{(\rho C_{p}{)}_{f}}. \end{array}\}$$ (16)

## 3. Physical quantities

The mathematical expression regarding skin friction coefficient [27] is given by

$$Cf_{x}=\frac{\tau }{\frac{U_{w}^{2}}{2}},$$ (17)

after utilizing the similarity transformation the non-dimensionless formula of drag force coefficient is

$$\frac{1}{2}Cf_{x}Re_{x}^{1/2}=g(0).$$ (18)

The expression regarding heat transfer Nusselt number [27] is

$$Nu_{x}=\frac{xq_{w}}{k_{f}(T_{0}-T_{\infty })},$$ (19)

whereas *q*_*w*_ indicates heat flux having expression mentioned below

$$q_{w}=-k_{nf}{\left(\frac{\partial T}{\partial y}\right)}_{y=0}+q_{r}.$$ (20)

The dimensionless Nusselt number after utilization of similarity transformation is manifested by

$$Nu_{x}Re_{x}^{-1/2}=-A_{2}\theta \prime (0),$$ (21)

## 4. Solution methodology

The MATLAB bvp4c which is the finite-difference scheme (collocation scheme) is executed to evaluate the non-dimensionless system of Eqs (12)–(14) sideways with boundary constraints (15). After allocating *f* = *w*_1_, *f*′ = *w*_2_, *f*′′ = *w*_3_, *θ* = *w*_4_, the system of ODEs are converted into first order, represented by

$$\begin{array}{l} w_{1}^{\prime }=w_{2},\\ w_{2}^{\prime }=\frac{w_{3}w_{3}^{2}+\gamma^{2}}{w_{3}^{2}+\lambda \gamma^{2}},\\ w_{3}^{\prime }=\frac{A_{1}}{3}w_{2}^{\prime^{2}}-\frac{2}{3}A_{1}w_{1}w_{2}^{\prime },\\ w_{4}^{\prime }=w_{5},\\ w_{5}^{\prime }=\frac{Pr\delta w_{1}w_{2}w_{5}-\frac{2}{3}A_{3}Prw_{1}w_{5})}{\left((A_{2}+\frac{4}{3}Rd)-Pr\delta_{1}xw_{1}^{2}\right)}, \end{array}\}$$ (22)

associated boundary conditions are

$$\begin{array}{l} \eta =0:w_{1}(0)=0,w_{2}(0)=1+\gamma_{1}\xi_{1},w_{4}(0)=1+\gamma_{2}\xi_{2},\\ \eta \to \infty :w_{2}(\infty )\to 0,w_{4}(\infty )\to 0. \end{array}\}$$ (23)

**Fig 3:**
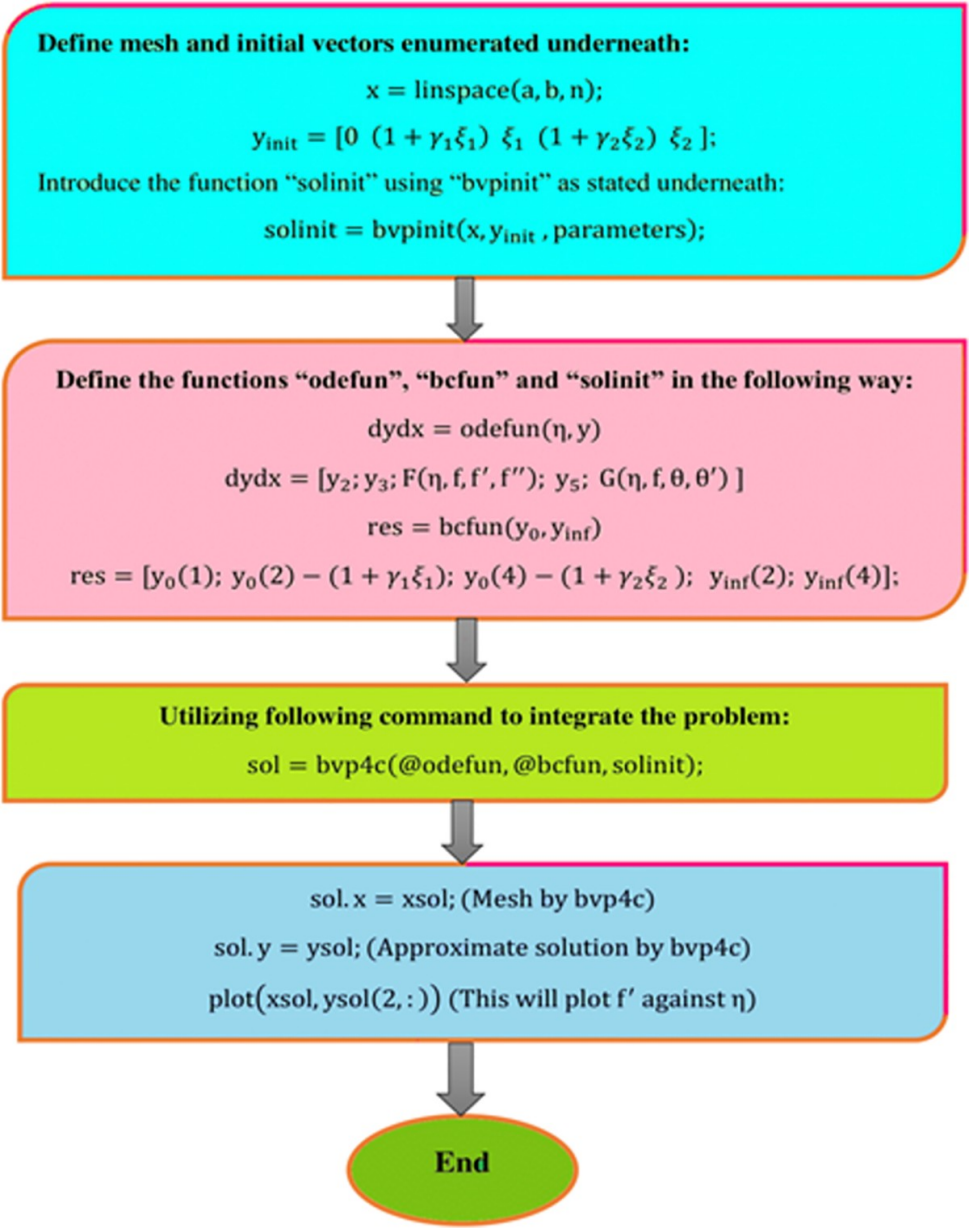
The bvp4c scheme’s flow chart method.

In the situation of the present problem, the tolerance rate and convergence value were set at 10^−6^ and *η*_∞_ = 7. Fig 3 is considered to illustrate the bvp4c numerical scheme’s flow chart mechanism.

## 5. Verification of code

The comparative analyses of the findings achieved with those stated by Reddy et al. [27] with the variation in *λ* and *γ* by holding other parameters fixed are given in Table 2. To obtain numerical calculations, the Matlab software bvp4c method is utilized. It is fully known from Table 2 that the findings obtained are very reliable and accurate.

**Table 2:** Study of the relation of present findings with Ref. [27] without *Rd*.

$Nu_{x}Re_{x}^{-1/2}$				
*γ*	*λ* = 0.5		*λ* = 1.0	
	Present	Ref. [27]	Present	Ref. [27]
0.1	0.130909	0.109782	0.144535	0.114058
0.2	0.109284	0.102621	0.144535	0.114058
0.3	0.085161	0.097438	0.144535	0.114058

## 6. Discussion

This sector is dedicated to studying the effect of sundry parameters that appear during the numerical simulation of the problem against shear stress field, velocity field, temperature field. Table 3 is sketched for the analysis of various dimensionless parameters on shear stress fields and the rate of heat transport for two kinds of CNTs. In the case of SWCNT, the domain of shear-stress *g*(*η*) increases because of an increment in fluid factor *λ* but *g*(*η*) lessens as a result of enrichment in Bingham number *γ* and velocity slip *γ*_1_ but no alteration is detected for the case of radiation *Rd*, Prandtl number *Pr*, thermal relaxation time parameter *δ*, and temperature slip parameter *γ*_2_. The rate of heat transport implies result in an improvement in *λ*, *Rd*, *δ* but heat transfer depreciates as a result of an improvement in the remaining parameters like *γ*, *γ*_1_, *γ*_2_, *Pr*. For the case of MWCNT, shear stress field *g*(*η*) augments owing to amplification in *λ* but decreases in the case of *γ* and *γ*_1_ and no change is obvious in the status of the remaining parameters. The number of Nusselt amplifies by the virtue of enrichment in the amounts of *Rd*, *λ* and *δ*. The Nusselt number depreciates as a result of an improvement in the remaining parameters like *γ*, *γ*_1_, *γ*_2_, *Pr*.

**Table 3:** Effect of distinct physical factors on the skin frictions and Nusselt number.

Physical parameters							SWCNT		MWCNT	
*γ*	*λ*	*γ*_1_	*Rd*	*Pr*	*δ*	*γ*_2_	*g*(*η*)	$Nu_{x}Re_{x}^{-1/2}$	*g*(*η*)	$Nu_{x}Re_{x}^{-1/2}$
0.5	0.1	0.1	0.1	24	0.01	0.1	0.3590	3.5454	0.3492	3.4368
0.7							0.2814	3.3449	0.2742	3.2533
0.9							0.2674	3.2413	0.2460	3.1585
1.1							0.2342	3.1713	0.2331	3.1099
	0.3						0.4734	3.6694	0.4629	3.5491
	0.5						0.5365	3.6983	0.5256	3.5752
	0.7						0.5821	3.7115	0.5709	3.5870
		0.3					0.3033	3.3963	0.2962	3.2991
		0.5					0.2661	3.2796	0.2605	3.1907
		0.7					0.2391	3.1843	0.2346	3.1018
			0.3				0.3590	3.3751	0.3492	3.2642
			0.5				0.3590	3.3931	0.3492	3.2812
			0.7				0.3590	3.4364	0.3492	3.3142
				25			0.3590	3.6227	0.3492	3.5104
				26			0.3590	3.6178	0.3492	3.5020
				27			0.3590	3.6009	0.3492	3.4816
					0.02		0.3590	3.5506	0.3492	3.4411
					0.03		0.3590	3.5557	0.3492	3.4453
					0.04		0.3590	3.5606	0.3492	3.4493
						0.3	0.3590	2.7954	0.3492	2.6880
						0.5	0.3590	2.3072	0.3492	2.2071
						0.7	0.3590	1.9642	0.3492	1.8721

Tables 4 and 5 are designed to study the effect of thermal radiation and velocity slip condition on Nusselt number and skin friction coefficient. From Table 4, it is quite clear that the heat transfer rate increases by increasing the thermal radiation parameter and maximum value at *Rd* = 2.5 which is 18.5%. From Table 5, it is quite evident the skin friction coefficient depreciates as a result of amplification in the velocity slip parameter and the maximum decrement is 67.9%.

**Table 4:** Heat transfer analysis in the presence/absence of thermal radiation.

Parameter	$Nu_{x}Re_{x}^{-1/2}$		$change=\left|\frac{Presence-Absence}{Presence}\right|\times 100\%$
*Rd*	Presence of *Rd*	Absence of *Rd*	
1	2.5099	2.2835	9.02%
1.5	2.6119	2.2835	12.6%
2	2.7094	2.2835	15.7%
2.5	2.8038	2.2835	18.5%

**Table 5:** Velocity slip impact on the skin friction coefficient.

Parameter	${Cf}_{x}Re_{x}^{1/2}$		$change=\left|\frac{Presence-Absence}{Presence}\right|\times 100\%$
*γ*_1_	Presence of *γ*_1_	Absence of *γ*_1_	
0.1	0.6284	0.6938	10.4%
0.3	0.5322	0.6938	30.3%
0.5	0.4642	0.6938	49.5%
0.7	0.4130	0.6938	67.9%

**Fig 4:**
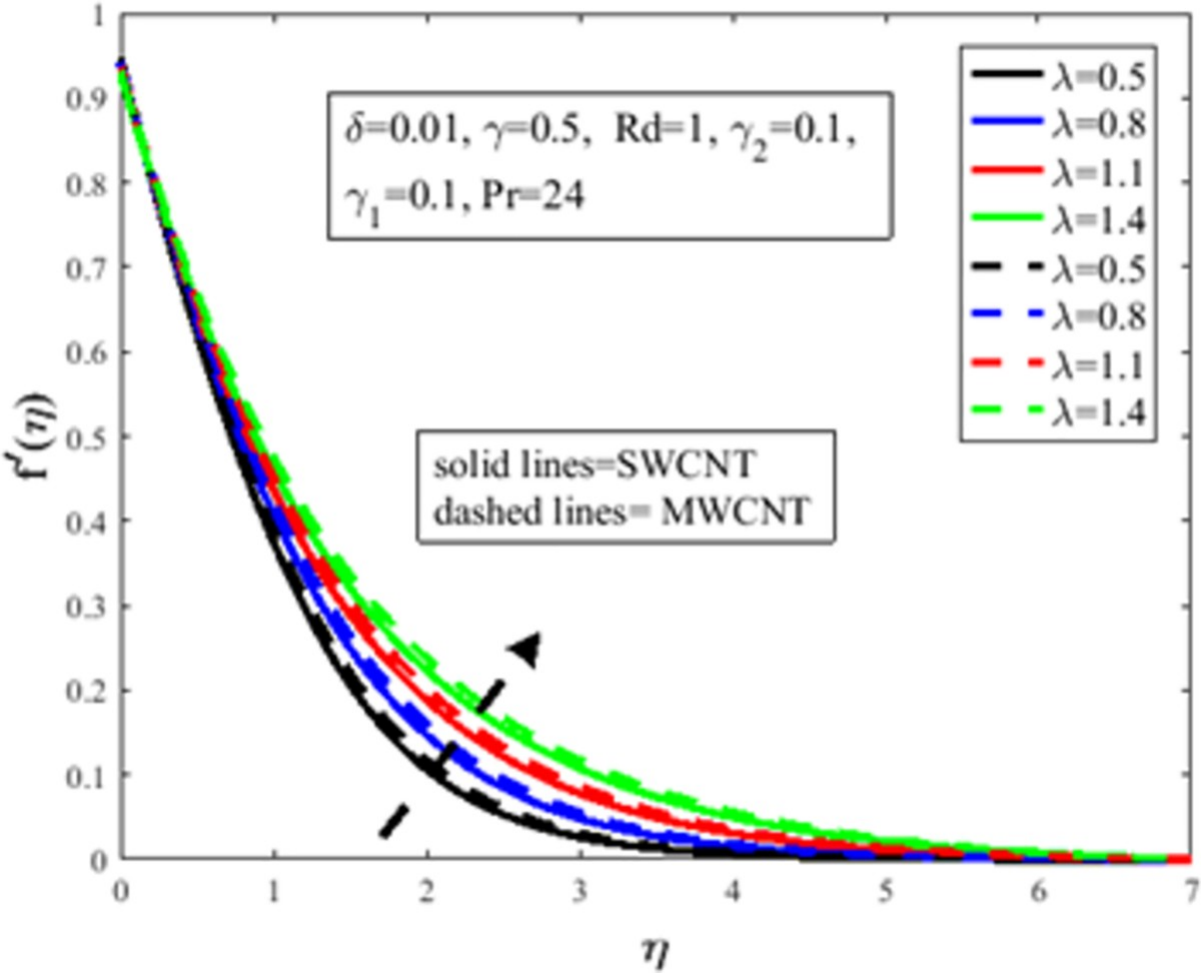
Effect of *λ* on *f*′(*η*).

**Fig 5:**
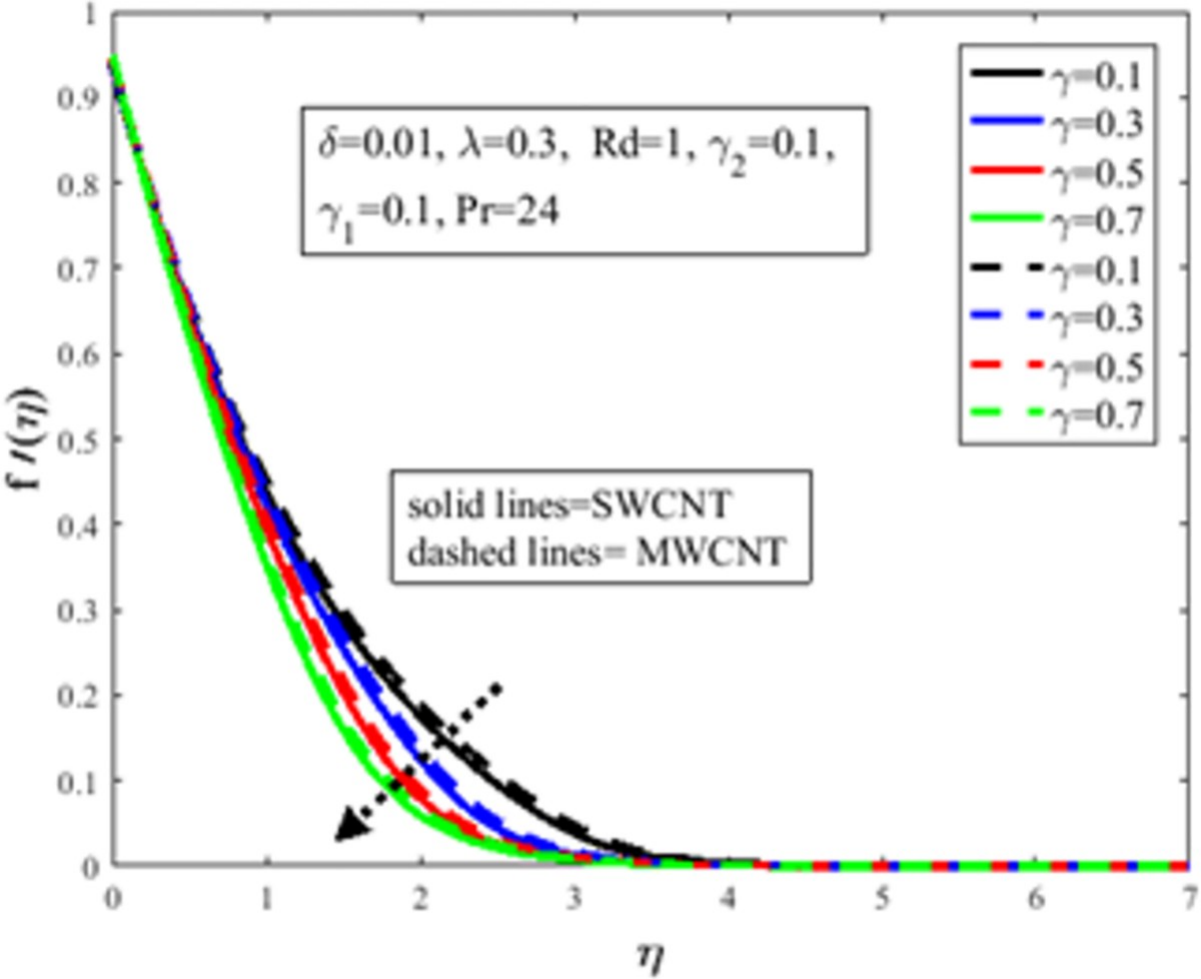
Effect of *γ* on *f*′(*η*).

**Fig 6:**
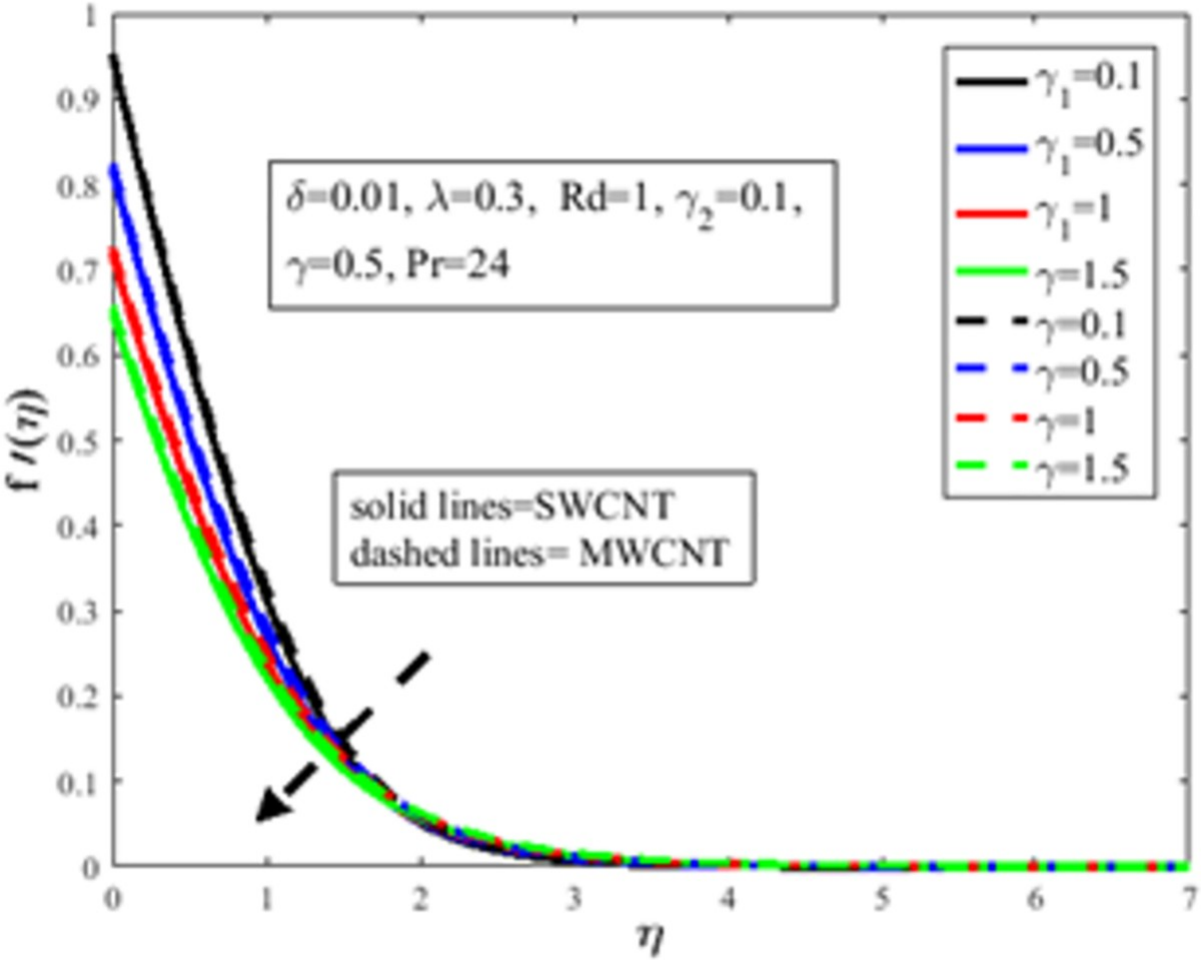
Effect of *γ*_1_ on *f*′(*η*).

**Fig 7:**
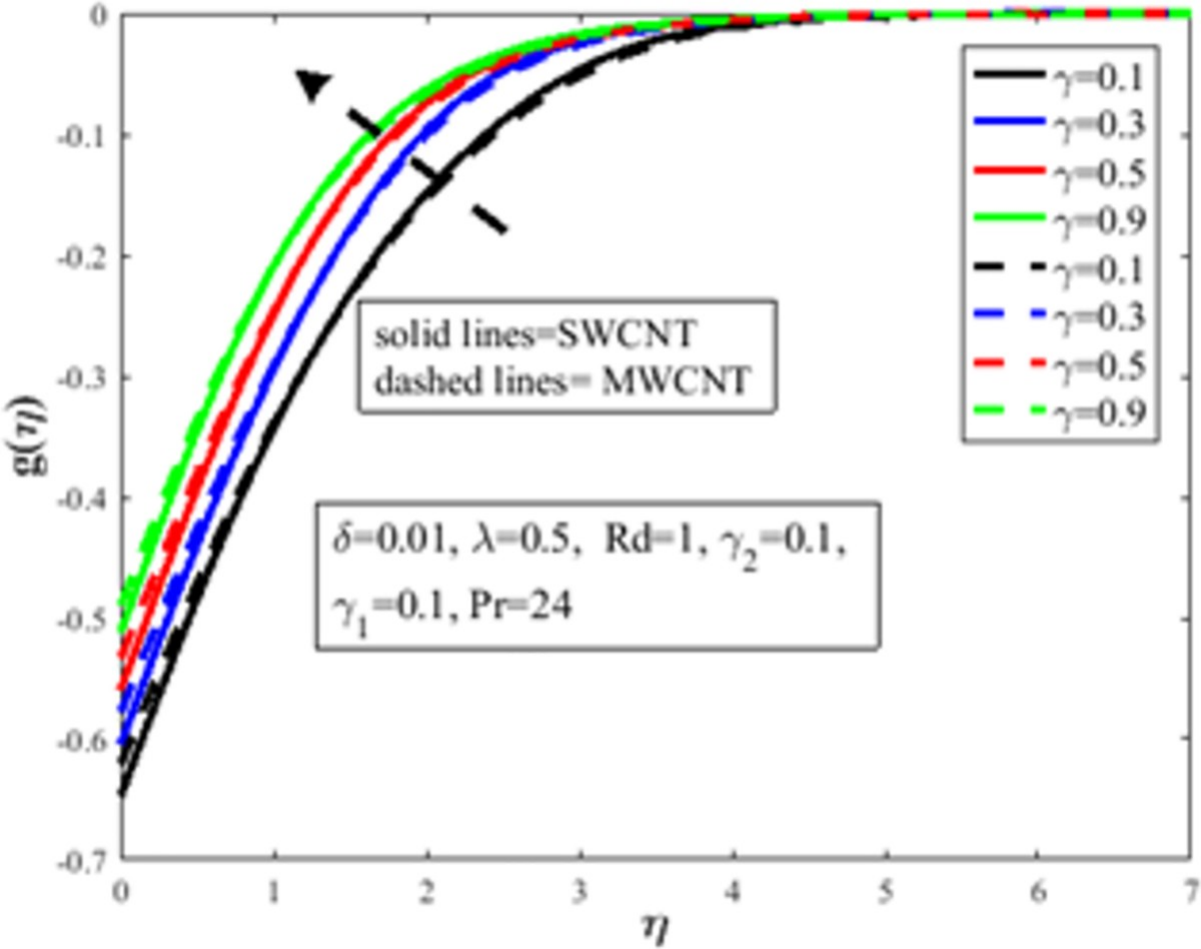
Effect of *γ* on *g*(*η*).

**Fig 8:**
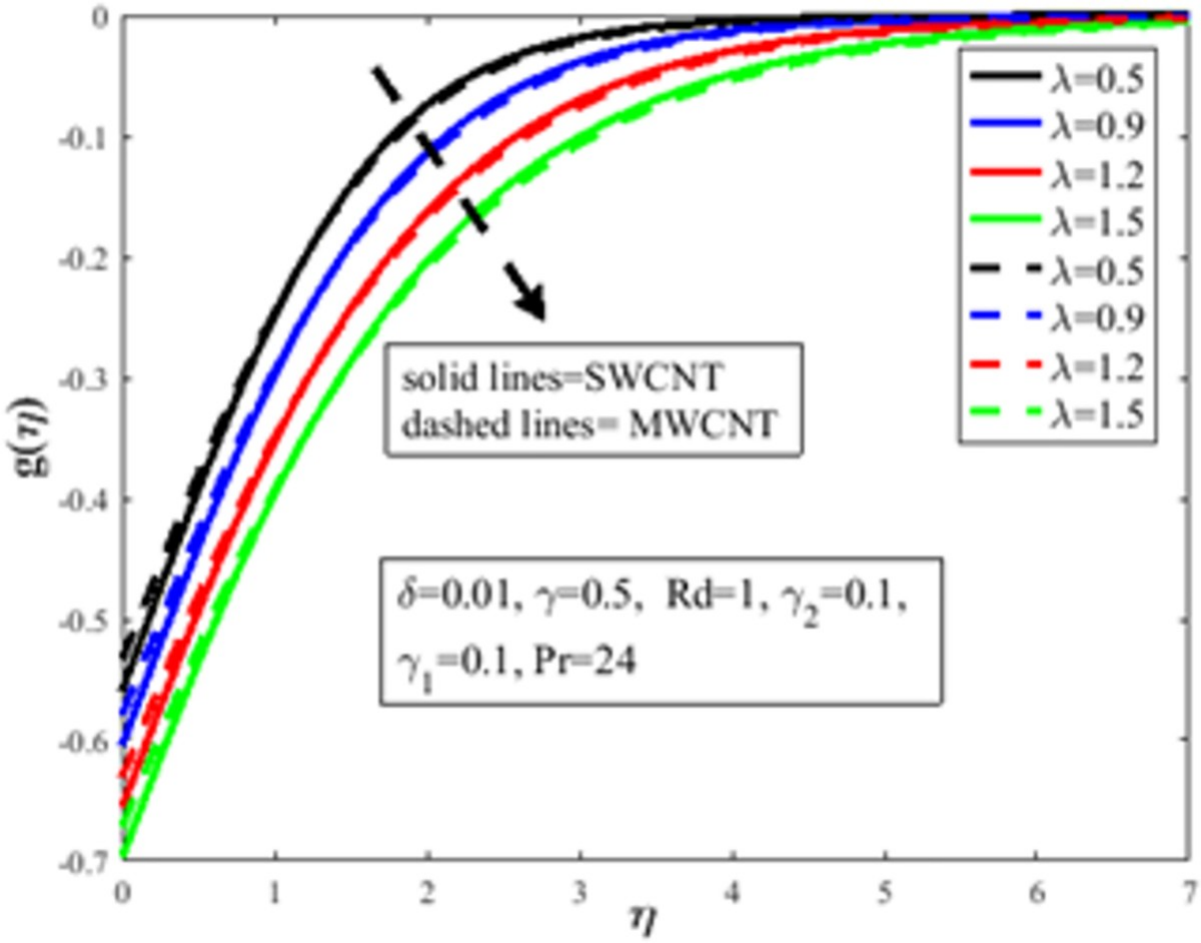
Effect of *λ* on *g*(*η*).

**Fig 9:**
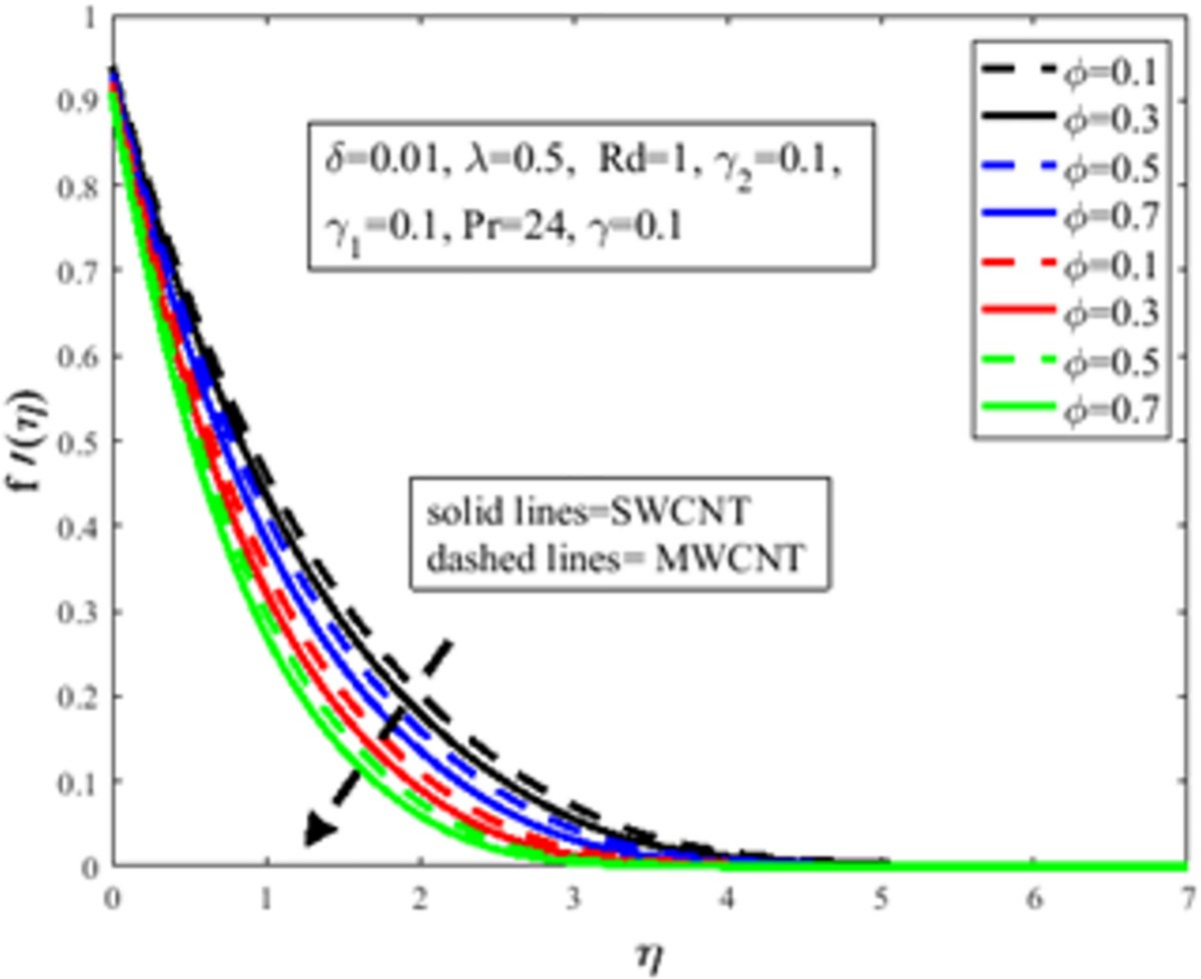
Effect of *ϕ* on *g*(*η*).

Fig 4 is designed to study the influence of RPF *λ* parameter on the profile of velocity *f*′(*η*). It is observed at an improvement in *λ*, the fluid behaves like shear thinning and the fluid flows more freely on the surface of the sheet which amplifies the velocity of the fluid and velocity profile. The Bingham number *γ* effect on the domain of velocity is represented in Fig 5. The proportion of yield-stress to viscous-stress is the number of Bingham. It is noted that due to an increase in the shear-rate, the fluid viscosity increases. Physically, amplification in Bingham turns liquid into solids due to magnification in the fluid viscosity. The fluid then behaves like shear-thickening, which often diminishes the velocity of the fluid. Fig 6 revealed the effect of velocity slip *γ*_1_ on *f*′(*η*). Slip phenomenon occurs when the velocity of the nanofluid and the surface is not the same. Physically, the slip phenomenon occurs due to roughness, wetness, and non-adhesives forces acting on the surface of the stretching sheet. That’s why they wander wall forces and adhesives forces are not acting on the fluid flow over the stretching sheet. As a result slip phenomenon occurs. In the presence of slip phenomenon, the nanofluid velocity flow over the surface declines which lessens the fluid velocity and *f*′(*η*). The portrayal of *γ* on shear stress field *g*(*η*) is shown in Fig 7. It’s noted that the shear stress field diminishes owing to amplification in *γ*. Physically for the fewer values of *γ* and *λ*, the fluid behaviour is shear thickening. Both *γ* and *λ* are prominent factors of the Reiner-Philippoff fluid parameter and are somehow related to each other. The behaviour of the fluid is shear-thinning *λ*<1 which lessens the fluid velocity and amplifies the stress field. As a result *g*(*η*) augments. Fig 8 depicts the outcome of *λ* on *g*(*η*). Fluid parameter *λ* is the ratio of zero shear rate to the infinite sheat rate. Physically a positive variation in the infinite shear rate depreciates the fluid parameter *λ*. The viscosity of fluid abates by the virtue of an amplification in *λ* which lessens the shear-stress of the nanofluid that furthermore leads to a decrement in the domain of shear-stress. Fig 9 is designed to reflect the effect of nanoparticles concentration *ϕ* on the profile of velocity. Physically concentration is proportional to the diffusivity. Molecules diffuse more quickly by the virtue of an increment in the concentration of nanoparticles. Concentration of the fluid increases due to an augmentation in the nanoparticles concentration of nanoparticles which lessens the fluid flow that eventually diminishes the velocity of nanofluid.

**Fig 10:**
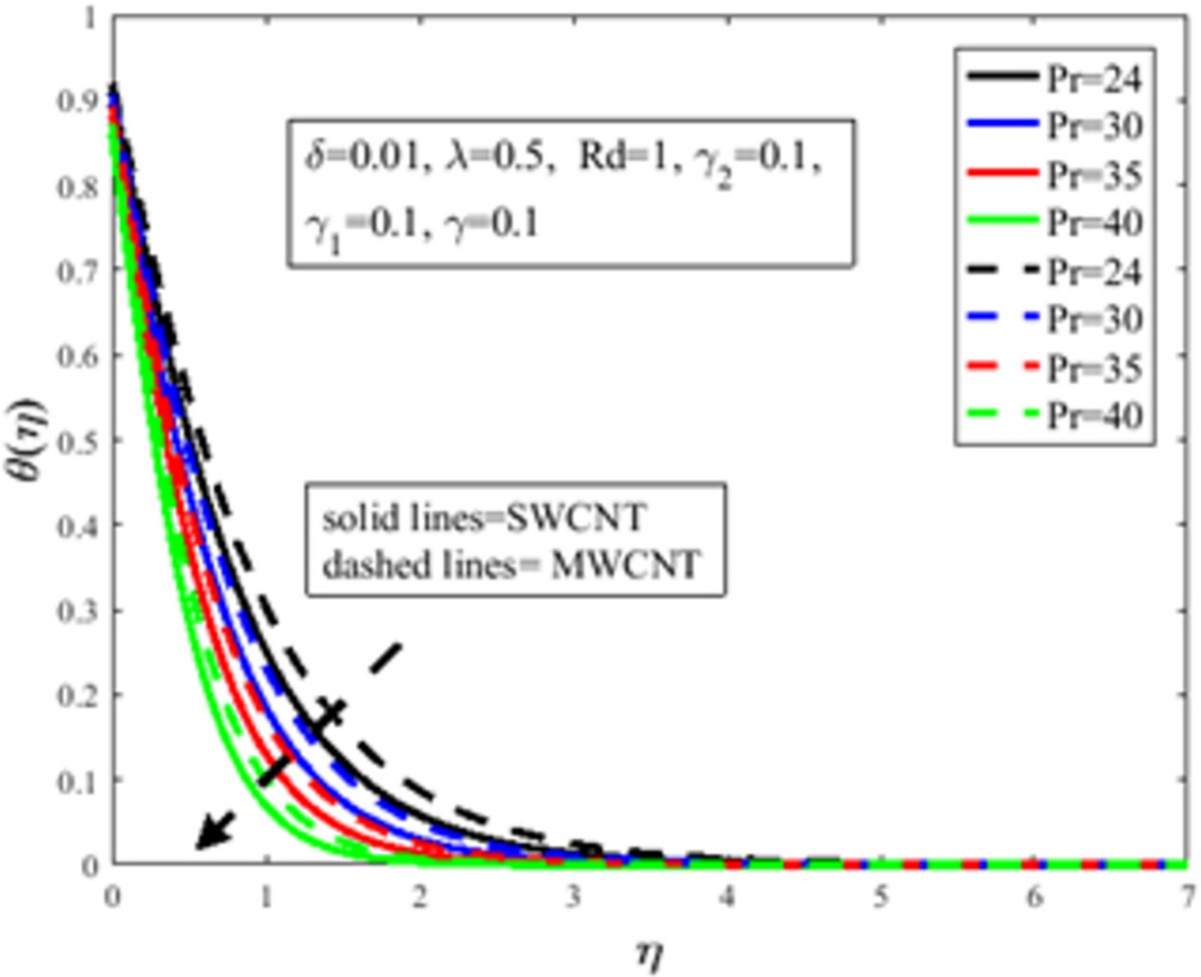
Effect of *Pr* on *θ*(*η*).

**Fig 11:**
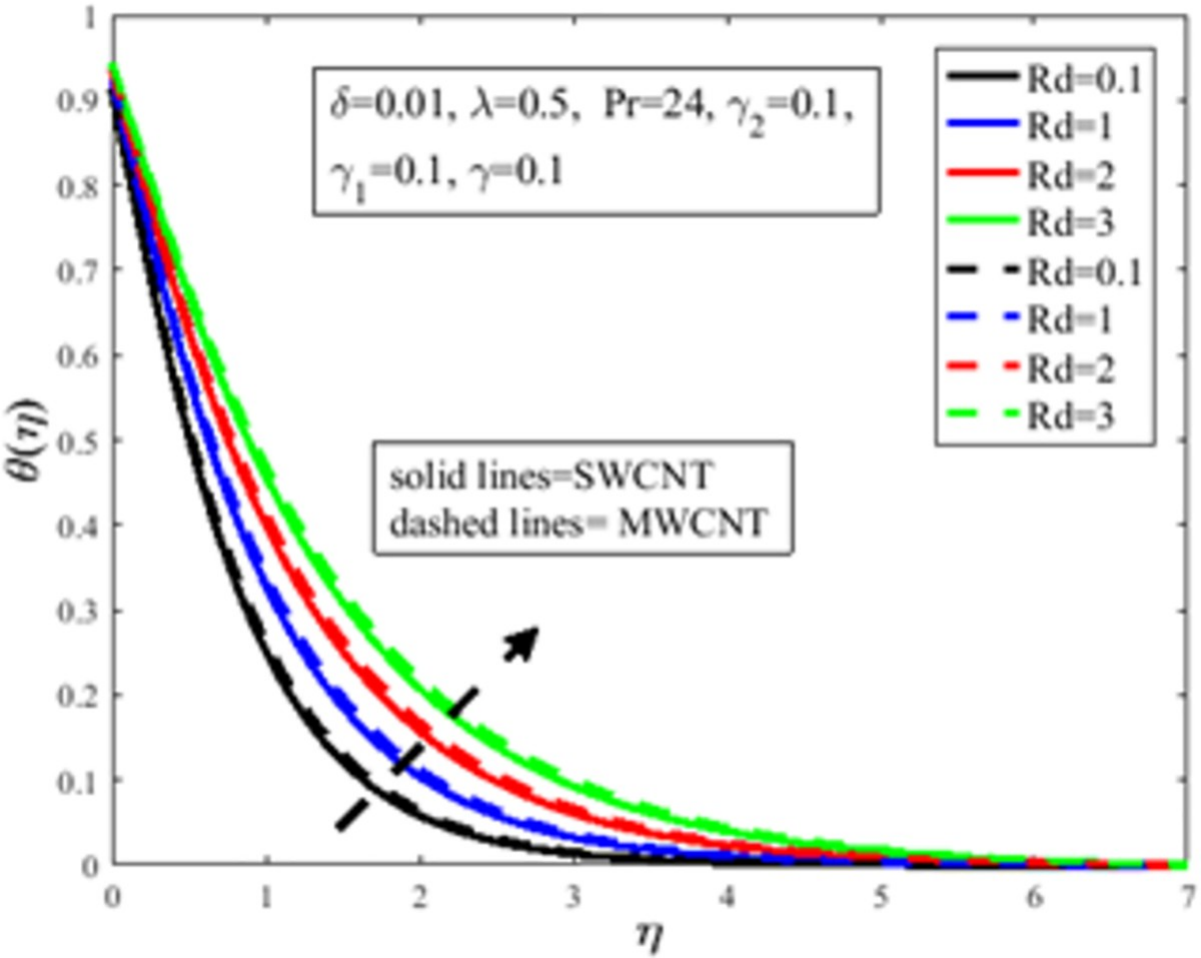
Effect of *Rd* on *θ*(*η*).

**Fig 12:**
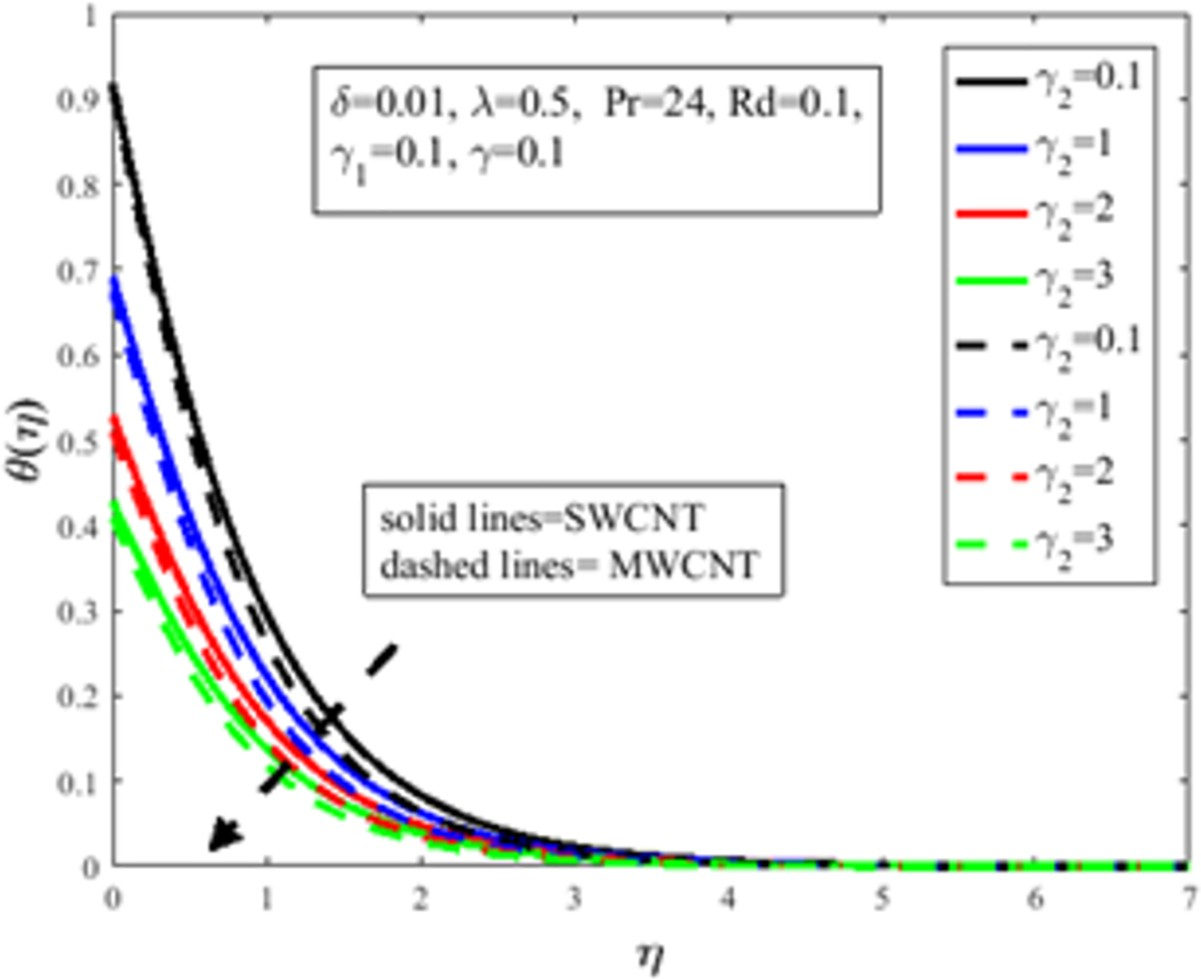
Effect of *γ*_2_ on *θ*(*η*).

**Fig 13:**
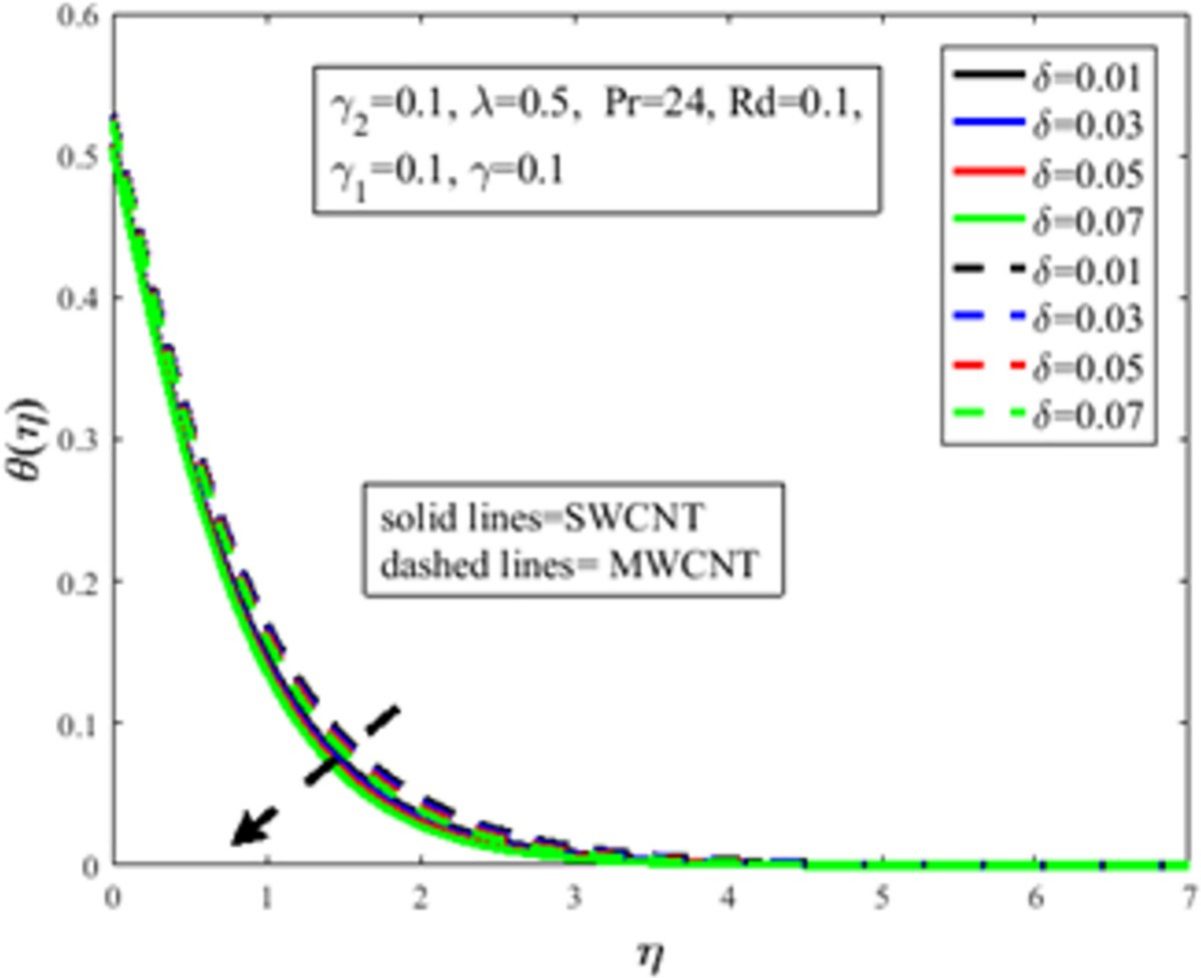
Effect of *δ* on *θ*(*η*).

**Fig 14:**
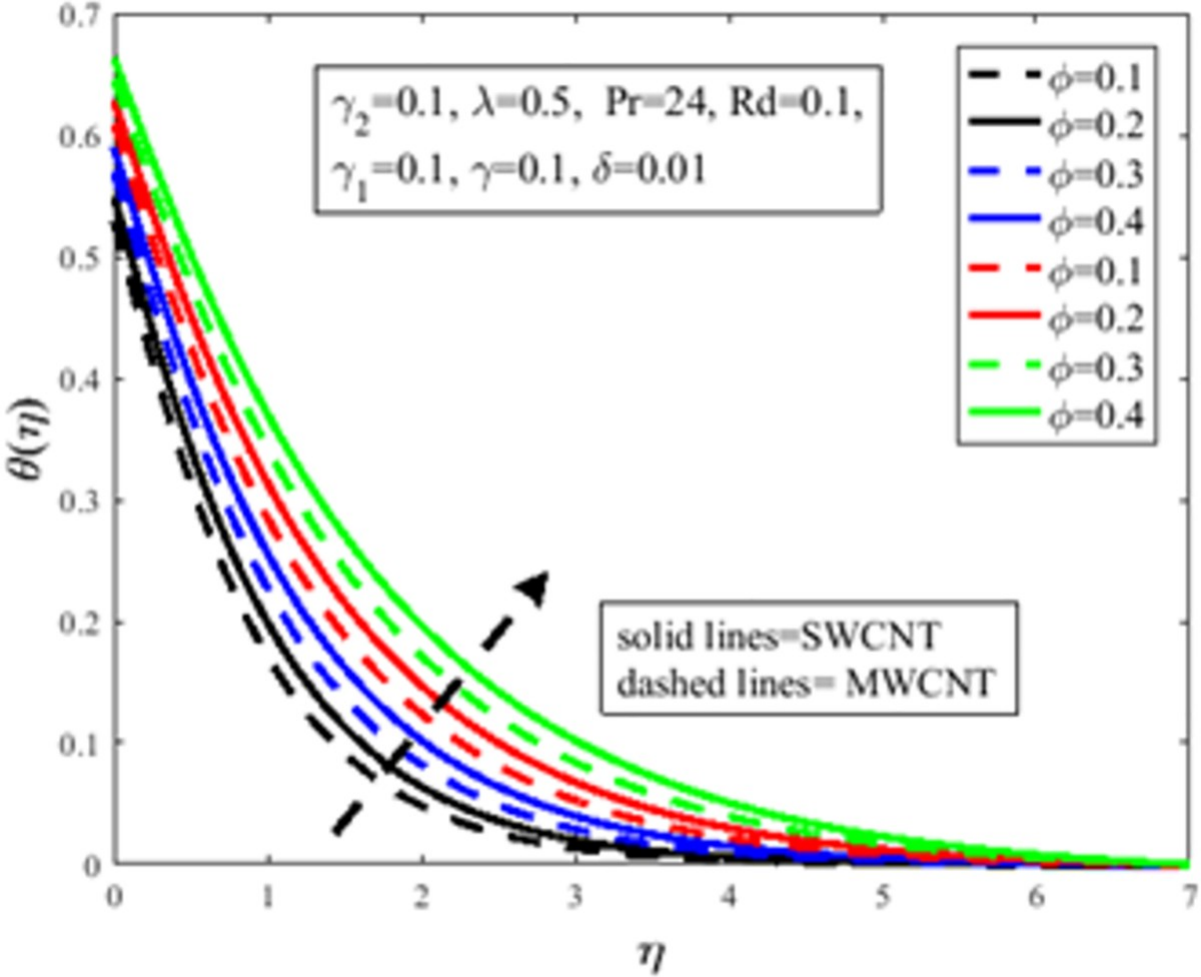
Effect of *ϕ* on *θ*(*η*).

In Fig 10, the Prandtl number *Pr* impact on the distribution of temperature field *θ*(*η*) is illustrated. The number of Prandtl is the proportion of momentum diffusion and thermal diffusion. Physically *Pr* is one of the prominent factors of boundary layer analysis. In the case of *Pr*<1, the thermal boundary layer dominates the momentum boundary layer but the situation is quite opposite in the case of *Pr*>1 where the momentum boundary layer dominates the thermal boundary layer. It is well established, the thermal diffusion of the fluid diminishes result in an increase in *Pr*. The temperature of the fluid depreciates as a result of an abatement in the thermal diffusivity which furthermore guides to a decrement in *θ*(*η*). The characteristics of radiation parameter *Rd* on *θ*(*η*) are highlighted in Fig 11. Physically thermal radiation is used to enhance the heat transfer rate of the fluid having immense utilization in an industry like polymer production, combustion reactors, pasteurization, and remove toxic microorganisms from the liquids. Sun is the best source of thermal radiation. Radiations enhance the nanofluid temperature. In the presence of *Rd* heat enter more easily into the system which improves the fluid temperature and temperature field *θ*(*η*). Fig 12 elucidates the influence of the temperature slip factor *γ*_2_ on the temperature profile *θ*(*η*). Physically temperature slip phenomenon occurs when the temperature of the sheet and fluid flow over the sheet is not in thermal equilibrium. With temperature slip condition, the temperature of the nanofluid declines which lessens the fluid temperature and *θ*(*η*). Fig 13 exhibits the parameter dependencies of thermal relaxation *δ* on the temperature field *θ*(*η*). Physically thermal relaxation time is the time in which fluid is allowed to relax to retain its original shape. During this time the fluid behaviour is shear thickening. As a result of a rise in *δ*, the nanofluid particles show nonconducting behaviour, which takes a long time to convey heat to their neighboring nearby particles. The temperature distribution diminishes as a consequence. Fig 14 illustrates the influence of nanoparticles concentration *ϕ* on *θ*(*η*). Physically it is quite evident that the insertion of nanoparticles in the base fluid enhances the thermal conductivity of the fluid and ultimately amplifies the heat transfer rate. The thermal conductivity of nanoparticles increases owing to an amplification in *ϕ* which moreover augments the temperature of fluid and *θ*(*η*).

## 7. Conclusion

In the present research, a computational heat transfer of Reiner-Phillipoff nanofluids over a flat surface is investigated under the impact of Maxwell velocity slip and Smoluchowski temperature slip on CNTs with modified Fourier theory. The concluding remarks from this study are given below

■ An improvement in the fluid parameter *λ* escalates the velocity field but the fluid velocity depreciates by the virtue of amplification in the Bingham number *γ*.
■ The shear-stress domain depressed results in enrichment in the fluid factor *λ*.
■ Velocity of the fluid lessens due to an increment in the concentration of nanoparticles.
■ An amplification in velocity slip *γ*_1_ and temperature slip *γ*_2_ depreciates velocity and temperature profiles.
■ A positive variation in a parameter of thermal relaxation *δ* brings about a decrement in the temperature distribution.
■ Temperature field escalates by the virtue of amplification in the radiation parameter *Rd*.
■ Rate of heat transport augments owing to an augmentation in the volume fraction of nanoparticles.

## Supporting information

S1 Nomenclature (DOCX)
