# Supplementary material for: Impact of Maxwell velocity slip and Smoluchowski temperature slip on CNTs with modified Fourier theory: Reiner-Philippoff model
Source: PLoS One. 2021 Oct 14;16(10):e0258367. doi: 10.1371/journal.pone.0258367 (PMC8516255; doi:10.1371/journal.pone.0258367)
Supplement: S1 Nomenclature — (DOCX) [file pone.0258367.s001.docx]

**S1 Nomenclature**

| $C$ | fluid concentration | $D_{B_{\infty}}$ | ambient Brownian diffusion coefficient |
| --- | --- | --- | --- |
| $C_{0}$ | concentration at wall | $T_{\infty}$ | ambient temperature $(K)$ |
| $C_{\infty}$ | limiting concentration | $U(x)$ | stretching velocity $\left( \frac{m}{s} \right)$ |
| $\gamma$ | Bingham number | $\lambda$ | fluid parameter |
| $Sc$ | Schmidt number | $q_{w}$ | surface heat flux $\left( \frac{W}{m^{2}} \right)$ |
| $Nu_{x}$ | Nusselt number | $Sh_{x}$ | Sherwood number |
| $\Pr$ | Prandtl number | $Rd$ | radiation parameter |
| $\theta_{w}$ | temperature ratio (*K*) | $q_{r}$ | radiative heat flux $\left( \frac{W}{m^{2}} \right)$ |
| $\tau$ | shear stress $\left( \frac{N}{m^{2}} \right)$ | $\tau_{s}$ | reference shear stress $\left( \frac{N}{m^{2}} \right)$ |
| $\mu_{0}$ | shear viscosity $\left( \frac{Kg}{m.s} \right)$ | $\mu_{\infty}$ | limiting viscosity $\left( \frac{Kg}{m.s} \right)$ |
| $\rho_{nf}$ | bionanofluid density $\left( \frac{Kg}{m^{3}} \right)$ | $\alpha_{nf}$ | bionanofluid thermal diffusion $\left( \frac{m^{2}}{s} \right)$ |
| $c_{p}$ | specific heat $\left( \frac{J}{K} \right)$ | $k_{nf}$ | bionanofluid thermal conductance $\left( \frac{W}{m.K} \right)$ |
| $\rho_{b}$ | density of the blood | $\rho_{g}$ | gold density $\left( \frac{Kg}{m^{3}} \right)$ |
| $(\rho C_{p})_{b}$ | blood heat capacity $\left( \frac{J}{K} \right)$ | $(\rho C_{p})_{g}$ | heat gold capacity $\left( \frac{J}{K} \right)$ |
| $k_{b}$ | blood thermal conductance $\left( \frac{W}{m.K} \right)$ | $k_{g}$ | thermal conductivity of gold $\left( \frac{W}{m.K} \right)$ |
| $\sigma^{*}$ | Stefan-Boltzman constant | $\kappa^{*}$ | absorption coefficient |
| $\delta_{1}$ | thermal relaxation | $\delta_{2}$ | solutal relaxation |
| $\gamma_{1}$ | velocity slip | $\delta_{2}$ | temperature slip $(K)$ |
| $\sigma_{*}$ | Stefen Boltzman constant $\left( \frac{W}{m^{2}.K^{4}} \right)$ | $k_{*}$ | mean absorption coefficient |
